# Neurotransmitter receptor-related gene signature as potential prognostic and therapeutic biomarkers in colorectal cancer

**DOI:** 10.3389/fcell.2023.1202193

**Published:** 2023-11-30

**Authors:** Linjie Zhang, Yizhang Deng, Jingbang Yang, Wuguo Deng, Liren Li

**Affiliations:** ^1^ Department of Colorectal Surgery, Sun Yat-sen University Cancer Center, Guangzhou, China; ^2^ State Key Laboratory of Oncology in South China, Collaborative Innovation Center for Cancer Medicine, Sun Yat-sen University Cancer Center, Guangzhou, Guangdong, China

**Keywords:** colorectal cancer, neurotransmitter receptor, TCGA, prognosis, biomarker, immune infiltration

## Abstract

**Background:** Colorectal cancer is one of the most common malignant tumors worldwide. A various of neurotransmitter receptors have been found to be expressed in tumor cells, and the activation of these receptors may promote tumor growth and metastasis. This study aimed to construct a novel neurotransmitter receptor-related genes signature to predict the survival, immune microenvironment, and treatment response of colorectal cancer patients.

**Methods:** RNA-seq and clinical data of colorectal cancer from The Cancer Genome Atlas database and Gene Expression Omnibus were downloaded. Neurotransmitter receptor-related gene were collected from publicly available data sources. The Weighted Gene Coexpression Network Analysis (WGCNA), Least Absolute Shrinkage and Selection Operator (LASSO) logistic regression, Support Vector Machine-Recursive Feature Elimination (SVM-RFE), and Random Forest (RF) algorithms were employed to construct the Neurotransmitter receptor-related gene prognostic signature. Further analyses, functional enrichment, CIBERSORTx, The Tumor Immune Single Cell Center (TISCH), survival analysis, and CellMiner, were performed to analyze immune status and treatment responses. Quantitative real-time polymerase chain reaction (qRT-PCR) assays were carried out to confirm the expression levels of prognostic genes.

**Results:** By combining machine learning algorithm and WGCNA, we identified CHRNA3, GABRD, GRIK3, and GRIK5 as Neurotransmitter receptor-related prognostic genes signature. Functional enrichment analyses showed that these genes were enriched with cellular metabolic-related pathways, such as organic acid, inorganic acid, and lipid metabolism. CIBERSORTx and Single cell analysis showed that the high expression of genes were positively correlated with immunosuppressive cells infiltration, and the genes were mainly expressed in cancer-associated fibroblasts and endothelial cells. A nomogram was further built to predict overall survival (OS). The expression of CHRNA3, GABRD, GRIK3, and GRIK5 in cancer cells significantly impacted their response to chemotherapy.

**Conclusion:** A neurotransmitter receptor-related prognostic gene signature was developed and validated in the current study, giving novel sights of neurotransmitter in predicting the prognostic and improving the treatment of CRC.

## Introduction

Colorectal cancer (CRC), encompassing both colon and rectal malignancies, is prevalent cancer affecting the digestive system, ranking as a leading cause of cancer-associated mortality and morbidity globally (Siegel et al., 2020). Despite a stabilized or decreased incidence in recent decades, attributed to enhanced screening practices, such as colonoscopic polypectomy, and alterations in risk factors, including a reduction in smoking and increased aspirin consumption, the 5-year survival rate remains unsatisfactory (Edwards et al., 2014; Favoriti et al., 2016; Brenner and Chen, 2018; Keum and Giovannucci, 2019). The widely utilized tumor-node-metastasis (TNM) system plays a crucial role in clinical decision-making for risk assessment and treatment planning. However, owing to the high molecular heterogeneity of CRC, patients with seemingly identical clinicopathological features may exhibit considerable variation in the risk of recurrence and death (Lin et al., 2021). Thus, the identification of effective methods for early diagnosis has become a focal point of research endeavors.

Emerging evidence has highlighted the significance of the nervous system in the pathogenesis of malignancies, with nerves being identified as a critical component of the tumor microenvironment (Zahalka and Frenette, 2020). And as important neural signaling messengers, studies have suggested that neurotransmitters and their receptors play a crucial role in tumor proliferation, angiogenesis, and metastasis, contributing to cancer development. For example, activation of the β2-adrenoceptor has been shown to promote tumor growth and angiogenesis through increased expression of vascular endothelial growth factor, metalloproteases 2, and metalloproteases 9, which further enhance angiogenic and metastatic processes in ovarian, lung, and breast cancers (Thaker et al., 2006). Furthermore, neurotransmitter receptors are widely expressed on the surface of immune cells and are regulated by their corresponding neurotransmitters, thus affecting tumor immune responses (Jiang et al., 2020b; Cervantes-Villagrana et al., 2020).

The role of neurotransmitter receptors in CRC is also complex, as noted in the literature. Li et al. (Li et al., 2021) demonstrated that the overproduction of 5-hydroxytryptamine overproduction promotes colitis-associated CRC progression by enhancing NLRP3 inflammasome activation. In addition, another study found that atropine and muscarinic receptor 3 blockers reduced tumor weight, volume, and enhanced antitumor immune responses by increasing infiltration of CD4^+^ and CD8^+^ T cells and significantly reducing PD-L1 expression in a CRC mouse model (Kuol et al., 2022). Gamma-Aminobutyric Acid Type B Receptor (GABABR) also plays a pivotal role in CRC progression. GABABR1, a central component of GABABR, shows significantly lower expression in tumor tissues than in non-tumor normal tissues. It impairs the migration and invasion of CRC cells by inhibiting EMT and the Hippo/YAP1 pathway (Wang et al., 2021). However, studies focusing on subtype characterization and risk signatures based on neurotransmitter receptor-related genes in CRC remain limited.

Herein, we conducted Weighted Gene Co-Expression Network Analysis (WGCNA) and machine learning to establish a reliable signature rooted in neurotransmitter receptor-related genes (NRGs). And its prognostic utility was systematically evaluated in CRC patients. Additionally, we revealed the importance of these gene signatures in the immune microenvironment of CRC. The associations of antineoplastic drugs with MRS were also explored. Overall, this study provides a research basis for exploring the potential pathogenesis of CRC and offers new ideas for treating this disease.

## Material and methods

### Data collection

**FIGURE 1:**
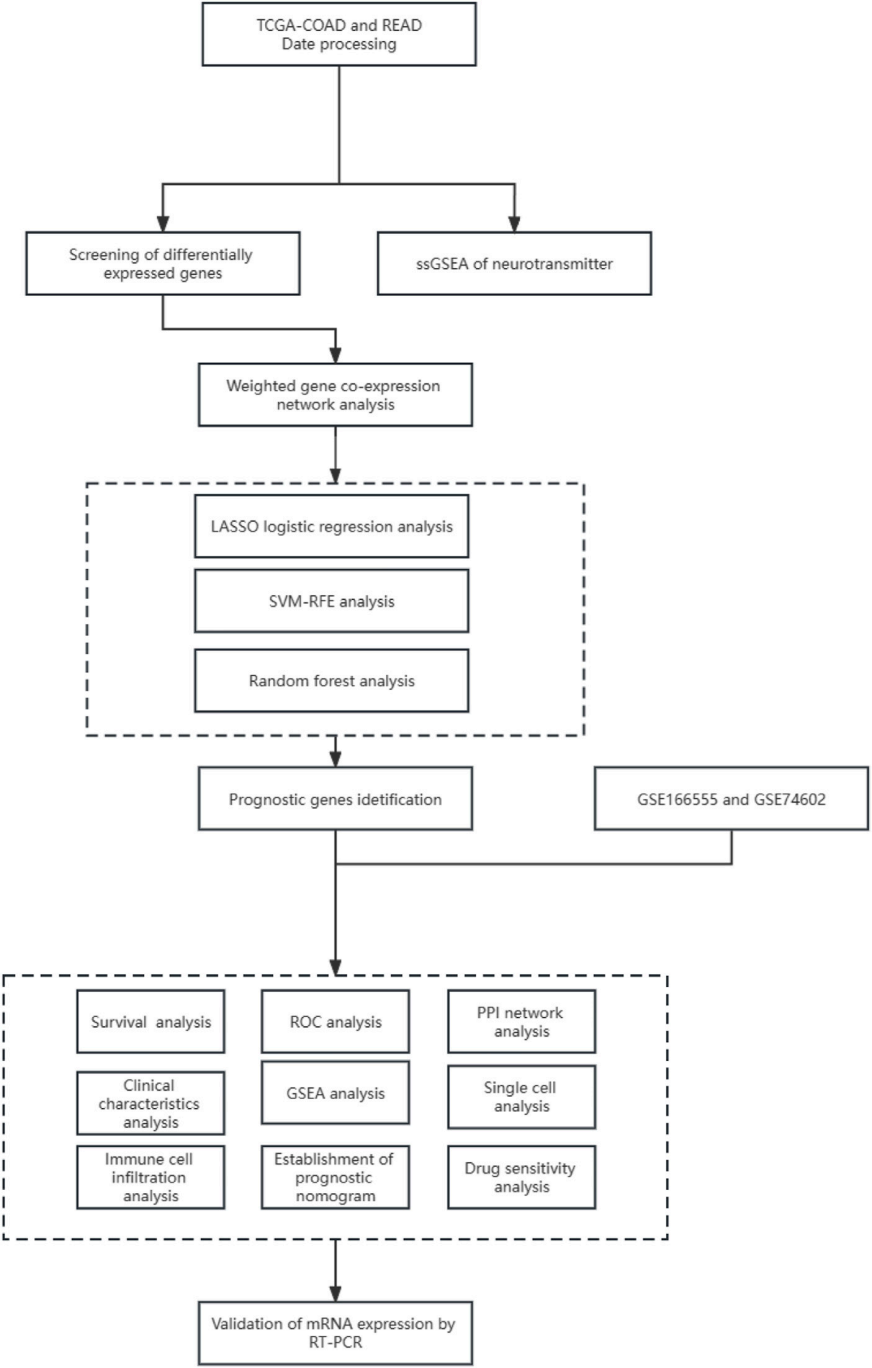
Schematic diagram of the workflow of the present study.

The workflow for this current study is presented in Figure 1. RNA sequencing and clinical data of 647 CRC cases were obtained from The Cancer Genome Atlas (TCGA) data portal, along with 51 normal tissue samples (https://portal.gdc.cancer. gov/). The microarray dataset GSE166555 and GSE74602 was downloaded from the Gene Expression Omnibus database (GEO, http://www.ncbi.nlm.nih. gov/geo/). Furthermore, the 114-NRGs list was obtained from the National Center for Biotechnology Information, United States National Library of Medicine (https://www.ncbi. nlm. nih.gov/gene/).

### Differentially expressed genes (DEGs) identification and survival Analysis

The TCGA sample was performed with DEGs analysis using the limma package (Ritchie et al., 2015). Survival analysis was conducted with the survivor R package, incorporating age to eliminate the impact of age on survival time. The results were visualized for COAD samples using the survminer R package. *p*-values were calculated using the Log-rank test.

### Weighted gene co-expression network analysis

The Weighted Gene Co-expression Network Analysis (WGCNA) method (Langfelder and Horvath, 2008; Li et al., 2023)was utilized to identify potential modules related to different subclusters of the DEGs expression matrix of TCGA. Abnormal samples were filtered out, and the Pearson correlation coefficient was calculated to construct the correlation adjacency matrix. Highly associated modules were selected for subsequent analysis. The intersection between the highly associated module and neurotransmitter receptor-related genes was estimated. Seven genes were present in both groups.

### Gene signature screening

Three machine learning algorithms were employed independently to screen diagnostic genes from the intersection, including Least Absolute Shrinkage and Selection Operator (LASSO) logistic regression (Zhao et al., 2020), Support Vector Machine-Recursive Feature Elimination (SVM-RFE) (Lin et al., 2012), and Random Forest (RF) (Guo et al., 2020). Genes that overlapped among these algorithms were considered diagnostic biomarkers, and their predictive utility was estimated using ROC curve analysis and the calculation of AUC values with the pROC package. The reliability and differential expression of the identified biomarkers were further confirmed in external testing cohorts. To investigate the expression of these genes in different stages of CRC patients, the clinical correlation analysis was utilized between the expression levels of these genes and CRC clinical characteristics. And the diagnostic genes were also analyzed with the PPI network using the STRING database (Chin et al., 2014).

### Functional enrichment analyses

Functional enrichment was performed using the Kyoto Encyclopedia of Genes and Genomes (KEGG) and Gene Ontology (GO) analyses with the ClusterProfiler and ggplot2 packages to interpret the biological effects of the intersection. Moreover, the prognosis genes was used to perform gene set enrichment analysis (GSEA) between subtypes through Java GSEA software and the results were visualized by the “enrich plot” R package.

### Analysis of immune cell infiltration

CIBERSORT was employed to calculate the immune cell content of each sample (Wang et al., 2023). We then used the Cor. test function to calculate the correlation coefficient between gene expression and immune cells. The correlation between gene expression and immune cells tested using Spearman’s correlation. Visualization of the results was performed using the ggpubr R package.

### Single cell analysis

The Tumor Immune Single Cell Center (TISCH) (http://tisch. comp-genomics. org/) was used to study the expression of the CHRNA3, GABRD, GRIK3, and GRIK5 gene in the tumor microenvironment as a single cell subset. TISCH is a scRNA-seq database that provides detailed annotations of cell types within the TME, allowing for exploring the TME in different cancer types (Sun et al., 2021).

### Construction of the prognostic nomogram

A nomogram was constructed using independent prognostic factors. Calibration curves were used to evaluate the performance of the nomogram. And decision curve analysis (DCA) was used to measure the net benefit of the nomogram.

### Drug sensitivity analysis

To investigate the relationship between prognosis genes expression and drug sensitivity, the study downloaded gene expression and drug sensitivity data from the CellMiner dataset. Drugs without clinical trials or FDA approval were removed. The correlation coefficient between the expression of prognosis genes and drug sensitivity was calculated using the cor. test function in R language, with correlation tests conducted. A *p*-value less than 0.05 was considered significant for the correlation between the target gene and drug sensitivity. A positive correlation between the expression of prognosis genes and drug sensitivity was indicated by a correlation coefficient greater than 0.

### RNA isolation and RT-qPCR assay

A total of 3 colorectal cancer specimens were obtained from the hospital specimen bank. And the Institutional Review Board of Sun Yat-Sen University Cancer Center approved this study (G2022-075-01). Total RNA was isolated using the RaPure Total RNA Micro Kit (R4012; Magen, Guangzhou, GD, China) followed by cDNA synthesis using the HiScript II Q RT SuperMix for qPCR kit (R223-01; Vazyme, Piscataway, NJ, United States). The qPCR assay was conducted using ChamQ SYBR qPCR Green Master Mix (Q311-02; Vazyme). The primers used were listed in Supplementary Table S2. Protein expression levels of GABRD were acquired from the HPA database through immunohistochemistry (IHC) staining and the utilization of downloaded IHC image data from the HPA database (Sjostedt et al., 2020).

### Statistical analysis

All data were analyzed and graphed using GraphPad Prism 6.0 and R software (version 4.0.5). The experimental data were presented as mean ± *s*.d. of three independent trials. The Wilcoxon signed-rank test was used to compare the differences between the two groups. A *p*-value less than 0.05 indicated statistical significance, and the significance levels were set at *p ≤ 0.05, **p ≤ 0.01, and ***p ≤ 0.001.

## Results

### Differential analysis between normal and tumor colorectal tissues

**FIGURE 2:**
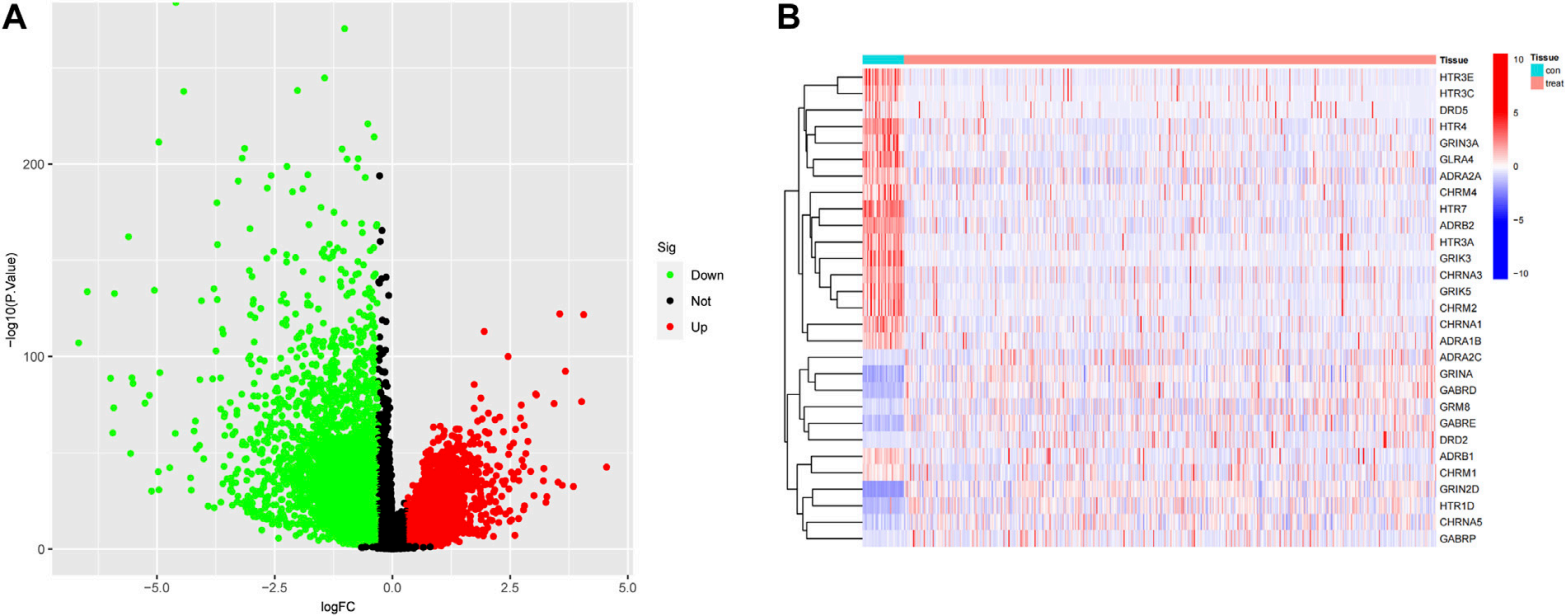
Identification of DEGs. **(A)** Differential Gene Expression Volcano Plot, where red indicates upregulation and green represents downregulation. **(B)** Neurotransmitter-related gene heatmap.

The volcano maps for TCGA DEG analysis are shown in Figure 2A. There was 29 neurotransmitter receptor-related DEGs in CRC compared with normal tissues, including 10 upregulated and 19 downregulated genes (Figure 2B).

### Gene modules derived from WGCNA based on DEGs expression of TCGA

**FIGURE 3:**
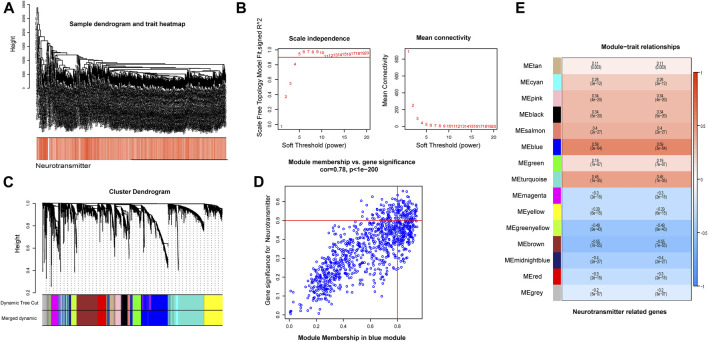
The co-expression modules analysis. **(A)** The samples were hierarchically clustered, and a clustering dendrogram was used to detect outliers. **(B)** Analysis of the scale-free fitting index (left) and average connectivity (right) used for selecting various soft-thresholding powers (β). **(C)** Clustering dendrogram of neurotransmitter-related genes; each color below represents a co-expressed gene module. **(D)** Scatter plot of key modules. Each point in the scatter plot represents a gene. **(E)** Heatmap describing the correlation between module and neurotransmitter score.

A co-expression network was constructed based on the DEGs expression matrix of TCGA (Figure 3A). We calculated a soft threshold and established a scale-free topology model with a data selection threshold of 5 (Figure 3B). After weight-based filtering, the cluster dendrogram was shown in Figure 3C. And the data were clustered into 17 modules. We then analyzed the correlation among the neurotransmitter scores of each module and found that the blue module had the strongest association with neurotransmitter scores (Figure 3E) (cor = 0.59 and P = 3e-64).

**FIGURE 4:**
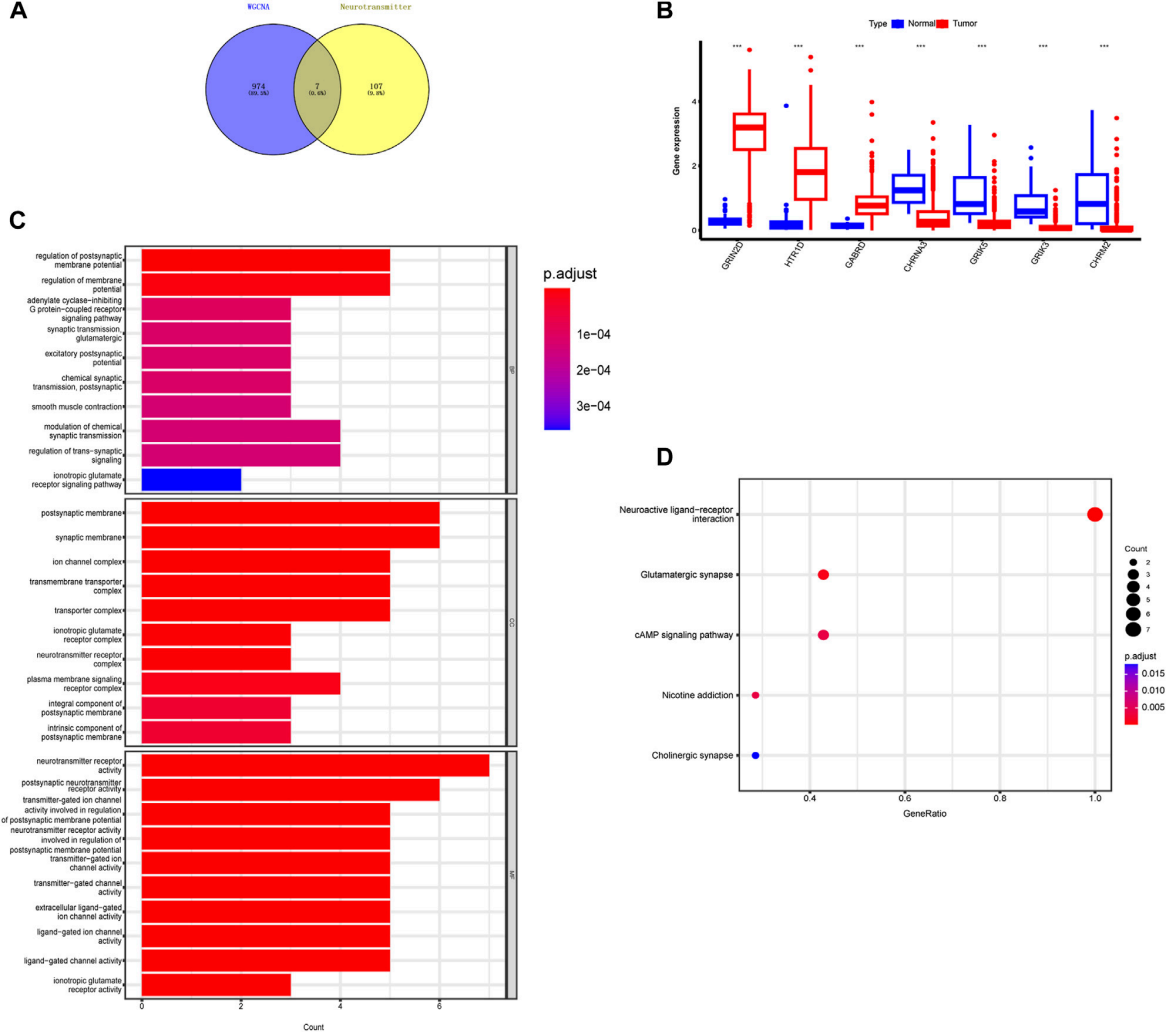
Identification of intersecting genes and functional enrichment analysis as well as expression of intersecting genes in CRC patients. **(A)** There are 7 intersecting genes between neurotransmitter-related genes and the blue gene module. **(B)** Expression of the 7 intersecting genes in colorectal cancer. **(C,D)** “GO enrichment analysis and KEGG enrichment analysis of the intersecting genes.

As a result, we selected the blue module as the crucial module for further analysis. A significant correlation existed between the blue modules’ MM and gene significance (GS) (Figure 3D). The intersection between the blue module and neurotransmitter receptor-related genes was also estimated (Figure 4A). Seven genes were identified as the candidate genes in both groups, including CHRNA3, CHRM2, GABRD, GRIK3, GRIK5, GRIN2D, and HTR1D. Except for GRIN2D, HTR1D, GABRD other genes are less expressed in tumors than in normal tissues (Figure 4B). In addition, GO and KEGG pathway analyses were performed on the candidate genes and revealed that these genes are mainly involved in the neurotransmitter receptor activity pathway (Figures 4C, D).

### Hub gene identification and verification

**FIGURE 5:**
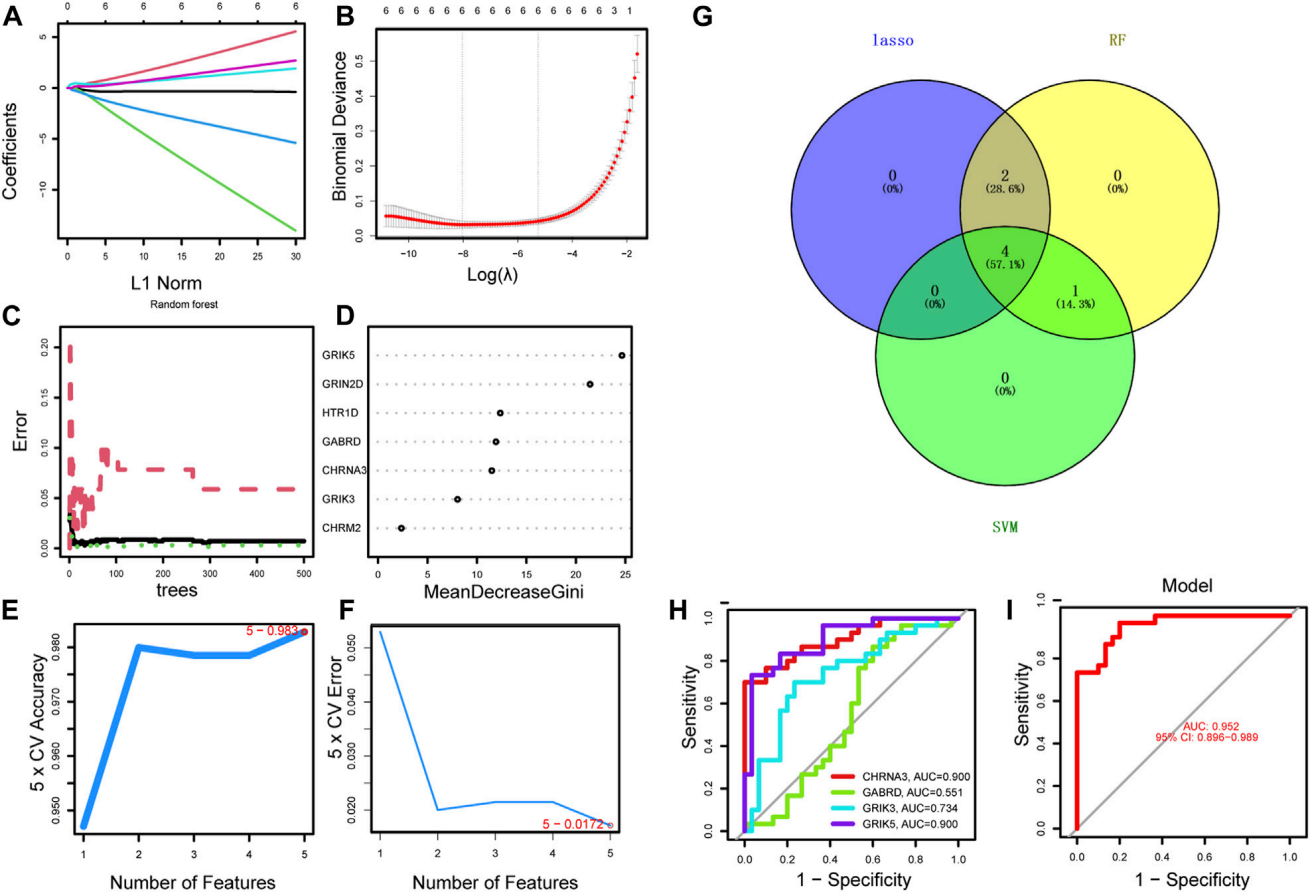
Identification of the diagnostic biomarkers from the intersecting genes. **(A)** Ten-fold cross-validation for tuning parameter selection in the LASSO model. Each curve corresponds to a gene. **(B)** LASSO coefficient analysis. The vertical solid line represents the standard error of the partial likelihood deviance. The vertical dotted line is drawn at the optimal λ. **(C)** Random forest for the relationship between the number of trees and the error rate. The minimum error was selected as the mtry node value, and the image value approaching stability was selected as the ntree value. **(D)** Ranking genes based on their relative importance. **(E–F)** SVM-RFE algorithm used for feature selection to narrow down the feature set and identify the most predictive feature genes. **(G)** The Venn diagram shows the intersection genes among LASSO, random forest, and SVM-RFE algorithms. **(H–I)** ROC curve to estimate the diagnostic performance of hub genes.

**FIGURE 6:**
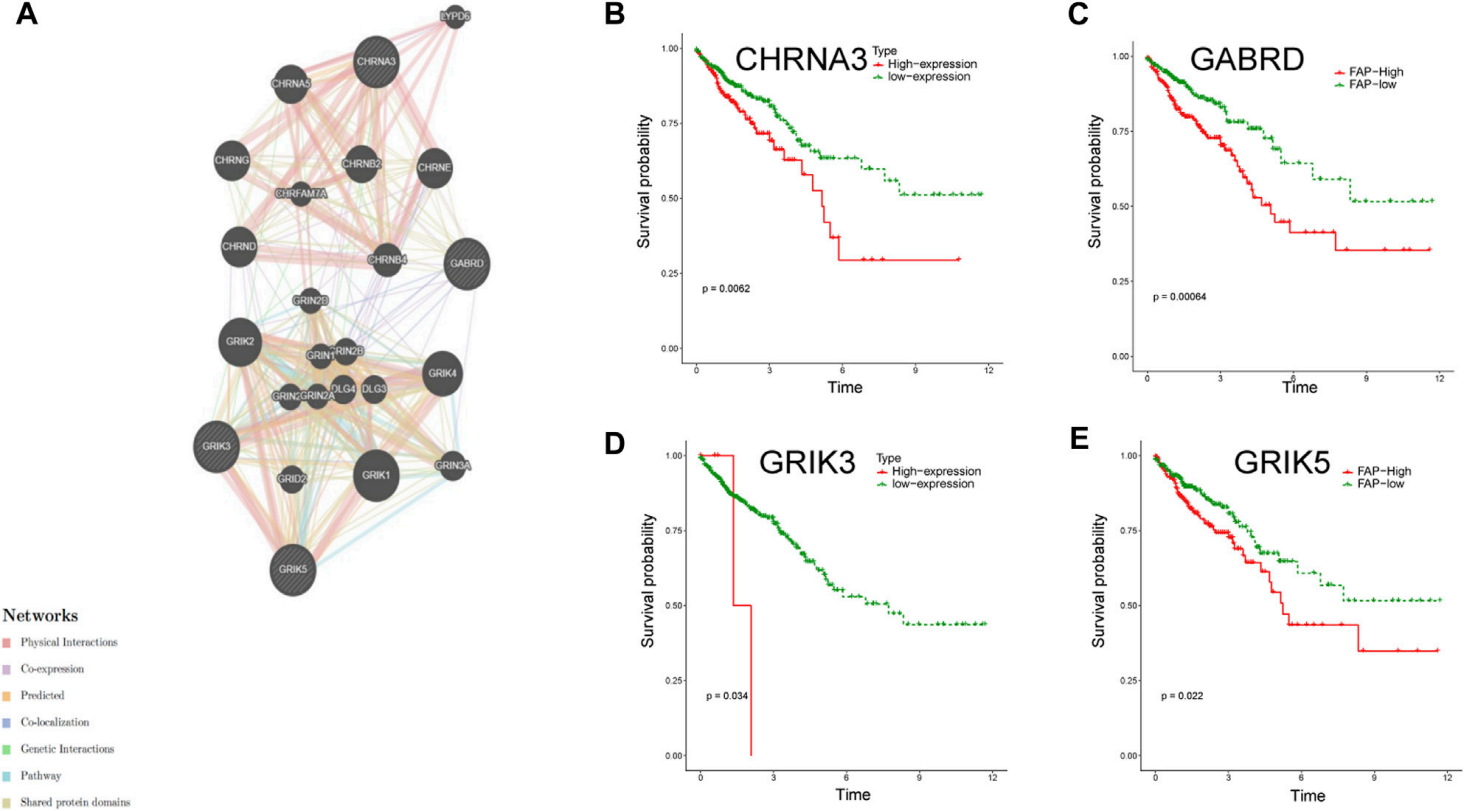
PPI and survival analysis of hub gene. **(A)** The GeneMANIA database performed PPI analysis on the diagnostic gene and its 6 interacting genes to predict correlations among co-localization, shared protein domains, co-expression, predicted and pathways. **(B–E)** Survival analysis of 4 hub genes.

Using the LASSO regression algorithm, six genes were identified as potential diagnostic biomarkers from the candidate genes (Figures 5A, B). RF identified five diagnostic genes (Figures 5C, D). All candidate genes were identified as potential biomarkers by the SVM-RFE algorithm (Figures 5E, F). Four genes (CHRNA3, GABRD, GRIK3 and GRIK5) were then overlapped via a Venn diagram, and served as robust diagnostic biomarkers (Figure 5G). The efficacy of these biomarkers was validated using the GSE74602 dataset, which showed high associated value with an AUC of 0.999 (Figures 5H, I). PPI analysis was also performed on the hub genes (Figure 6A). Finally, we explored the relationship between hub genes and clinical features and found that patients with advanced TNM stages had higher expression of the identified hub genes (Supplementary Figure S1). And high expression of CHRNA3, GABRD, and GRIK5 are significantly associated with poor prognosis (Figures 6B–E).

### GSEA analysis

**FIGURE 7:**
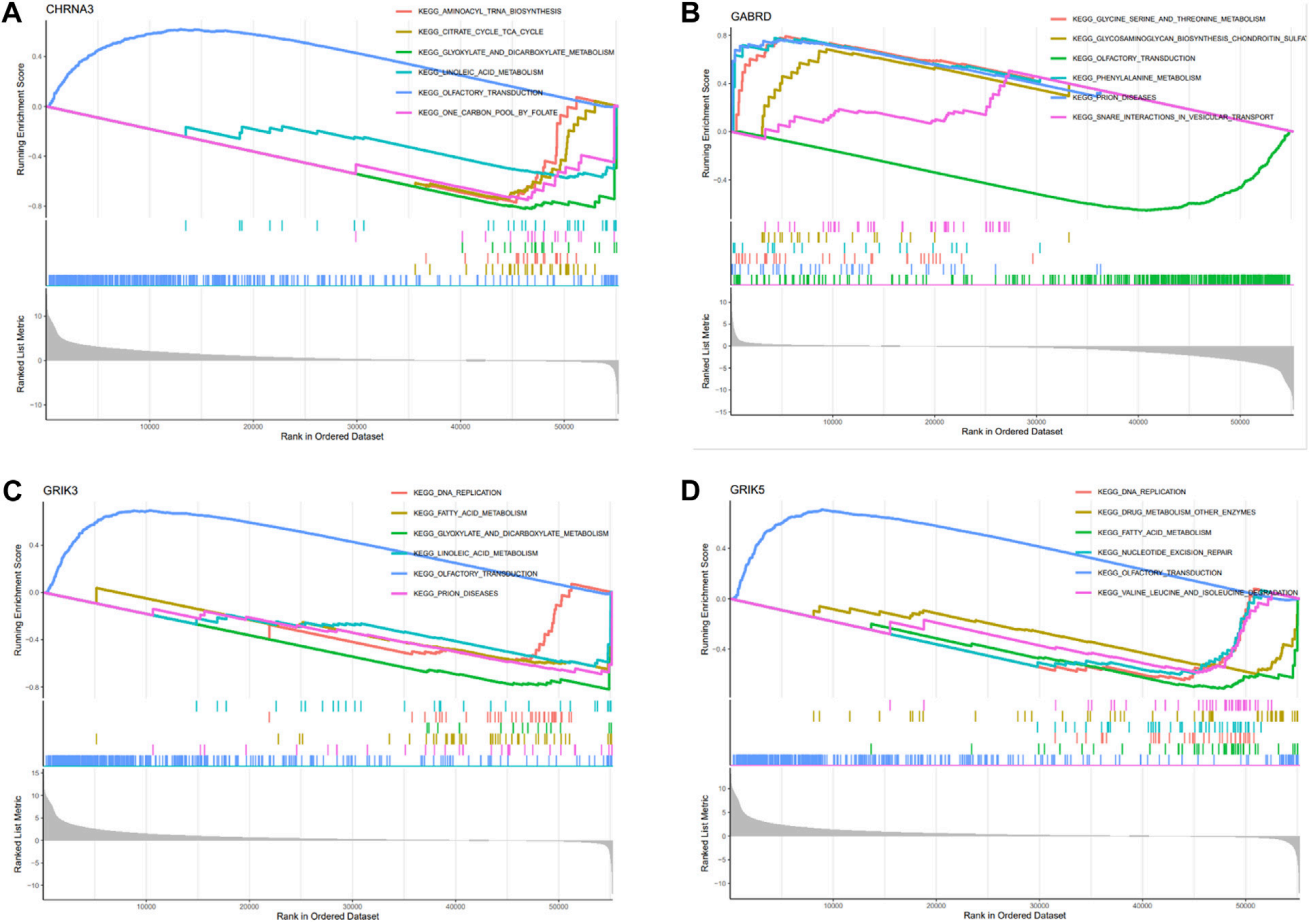
Enrichment analysis of hub genes. **(A–D)** Functional analysis of the 4 hub genes using GSEA.

We conducted GSEA analysis to investigate the signaling pathways associated with hub genes, and the top six pathways are presented in Figure 7. Our findings revealed that CHRNA3 was significantly linked to several pathways, including aminoacyl-tRNA biosynthesis, citric cycle/TCA cycle, glyoxylate and dicarboxylic acid metabolism, olfactory transduction, and one-carbon pool by folate (Figure 7A). The expression of GABRD was significantly associated with pathways related to glycine, serine, and threonine metabolism, glycosaminoglycan biosynthesis (chondroitin sulfate), olfactory transduction, phenylalanine metabolism, prion diseases, and SNARE interactions in vesicular transport (Figure 7B). The expression of GRIK3 was significantly correlated with pathways related to DNA replication, fatty acid metabolism, glyoxylate and dicarboxylic acid metabolism, linoleic acid metabolism, olfactory transduction, and prion diseases (Figure 7C). Lastly, the expression of GRIK5 was significantly linked to pathways related to DNA replication, drug metabolism (other enzymes), fatty acid metabolism, nucleotide excision, olfactory transduction, as well as valine, leucine, and isoleucine degradation (Figure 7D).

### Association of hub genes levels with tumor microenvironment

**FIGURE 8:**
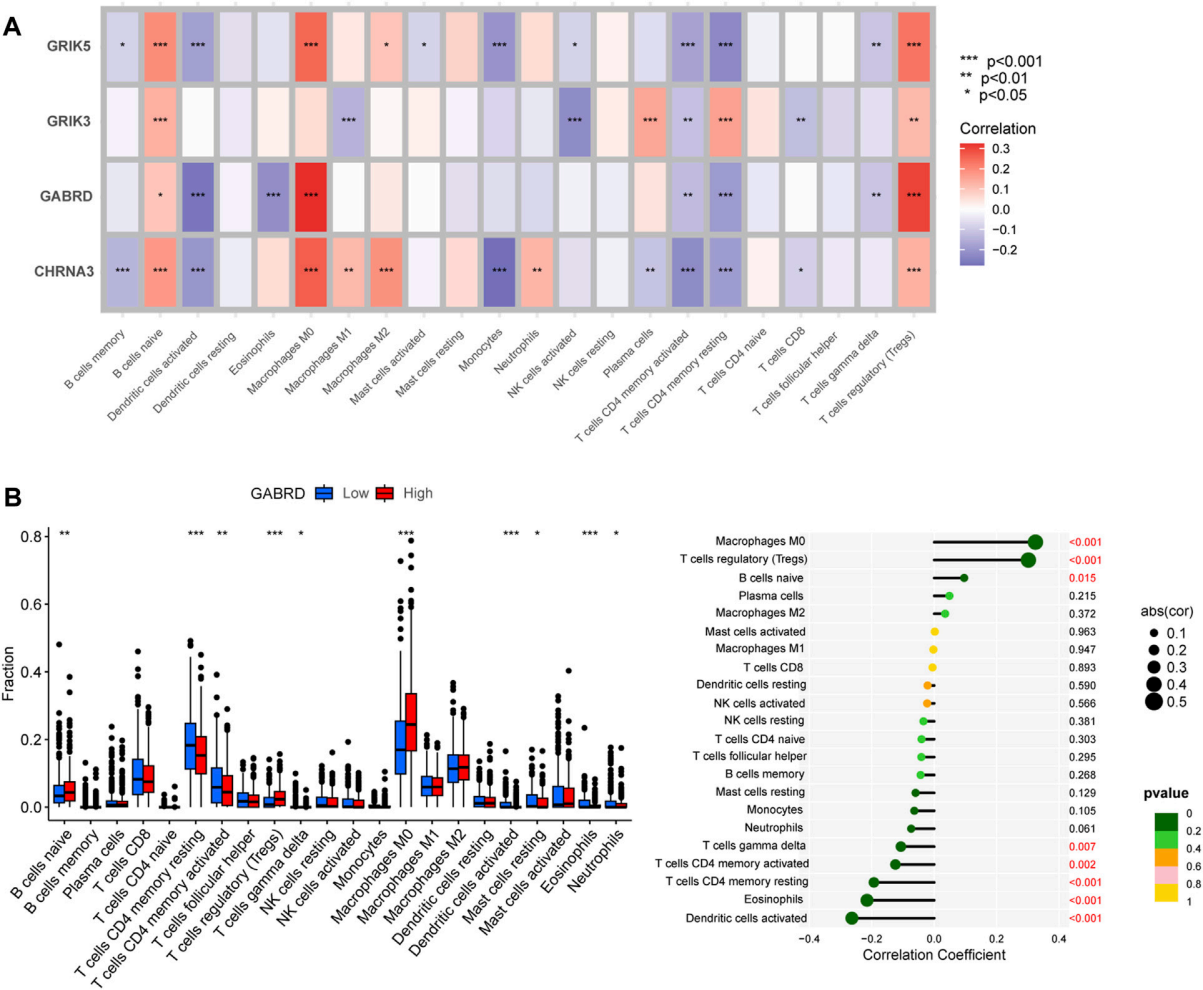
Association of hub genes with immune cell infiltration. **(A)** Correlation analysis between immune cells and hub genes. **(B)** Correlation analysis between GABRD and immune cells, as well as differences in immune cells between high and low gene expression groups.

**FIGURE 9:**
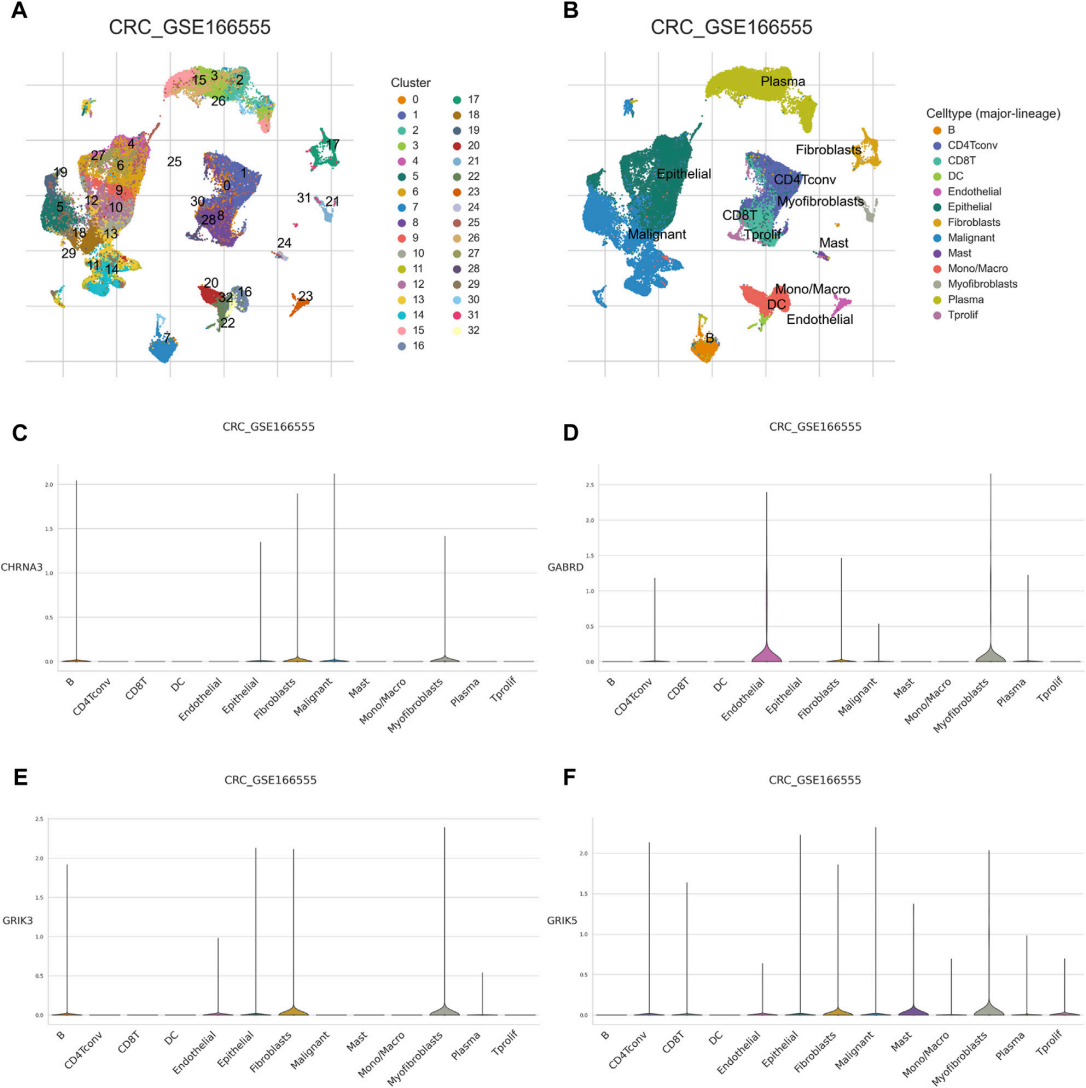
Single cell analysis of hub genes **(A)** Using the colorectal cancer GSE166555 dataset for study, dimensionality reduction and clustering analysis were performed. All cells were clustered into 32 clusters. **(B)** Cell annotation. A total of 13 cell types were identified. **(C–F)** expression of the 4 hub genes in single-cell data.

The Tumor Microenvironment (TME) consists of various components including endothelial cells, cancer-associated fibroblasts (CAFs), myofibroblasts, immune cells, and other factors (Ding et al., 2022). To investigate whether hub gene would be involved in TME, we observed the correlation of hub genes expression with stromal cell and immune cell infiltrations (Figure 8A, Supplementary Figure S2). The results showed that Tregs, naive B cells, and activated memory CD4^+^ T cells were positively correlated with all hub genes, while activated memory CD4^+^ T cells were negatively correlated. Macrophages M0 were found to be highly infiltrated in cases with high expression of CHRNA3, GABRD, and GRIK5. Only GRIK3 was positively correlated with the infiltration of resting memory CD4^+^ T cells (Figure 8B). Moreover, we found that CHRNA3, GABRD, GRIK3, and GRIK5 were also expressed in both endothelial and stromal cell subpopulations (Figures 9A, B). Notably, CHRNA3, GRIK3, and GRIK5 were highly expressed in fibroblasts compared to other cell subpopulations. And GABRD had high expression levels in endothelial cells, suggesting its role in angiogenesis within the TME (Figures 9C–F).

### Establishment of a prognostic nomogram for colorectal cancer

**FIGURE 10:**
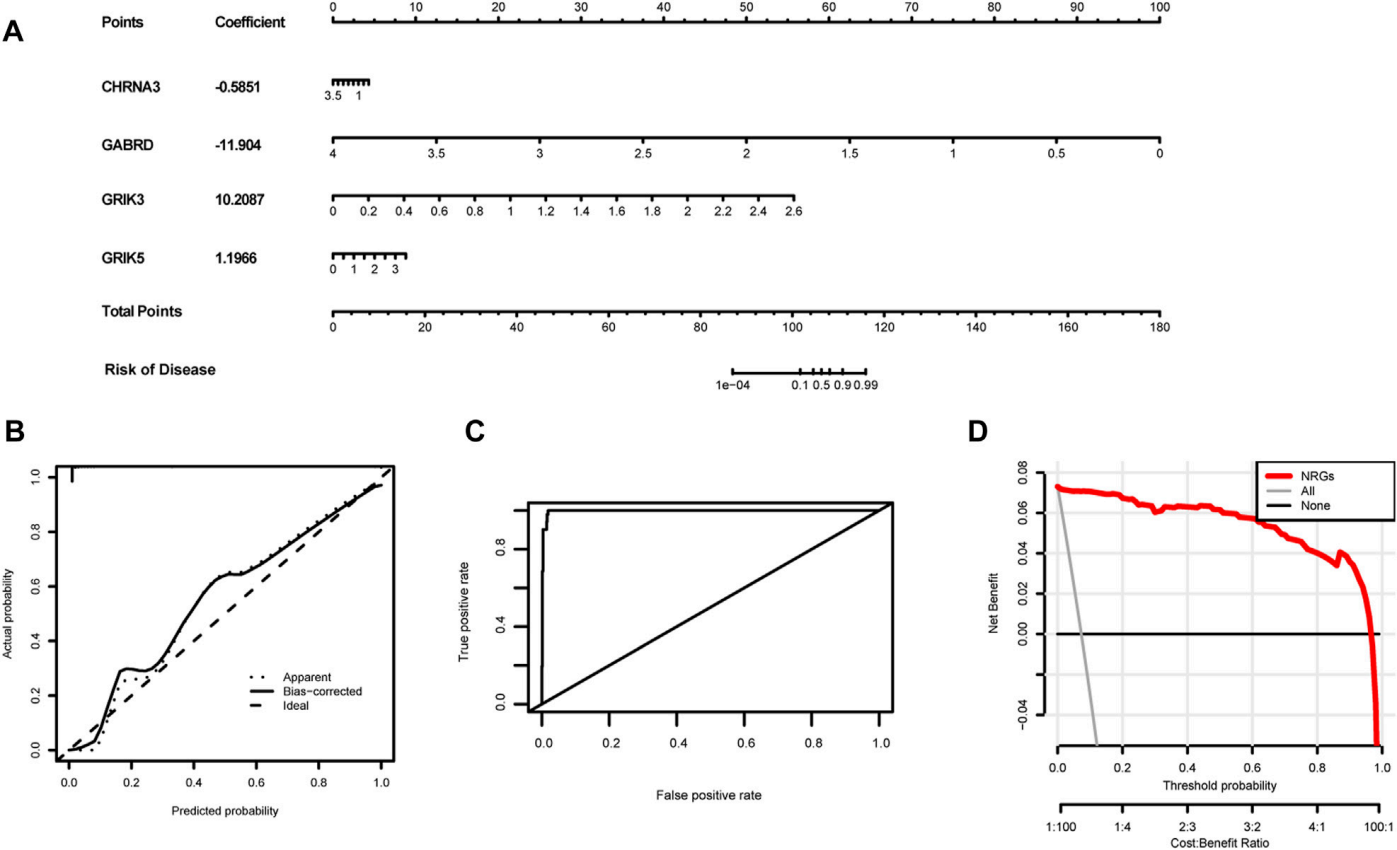
Establishment of the diagnostic nomogram. **(A)** Create a column chart of integrated hub genes, where each variable corresponds to a score that can be added together to calculate a total score, to predict the progression of colorectal cancer. **(B)** Calibration curves were used to estimate the predictive accuracy of the column chart. **(C)** Harrell’s concordance index was used as performance metrics. **(D)**Decision curve analysis shows the clinical benefit of the nomogram for predicting the progression of colon cancer.

We developed a novel prognostic nomogram to offer a reliable and quantifiable method for predicting the progress of colorectal cancer based on the hub gene. In the nomogram, each hub gene is assigned a score, calculated by multiplying the gene’s coefficient with its expression level. And the total score, determined by summing the scores of all the hub genes, corresponds to varying risk levels for patients (Figure 10A). In addition, calibration curves and Harrell’s concordance index (C-index) showed the nomogram had good predictive power (Figures 10B, C). Subsequently, Decision curve analysis elicited that the nomogram provided a significant net benefit (Figure 10D). Overall, these results indicate that the nomogram possesses significant predictive value.

### Expression of genes with sensitivity of cancer cells to anti-tumor drugs

**FIGURE 11:**
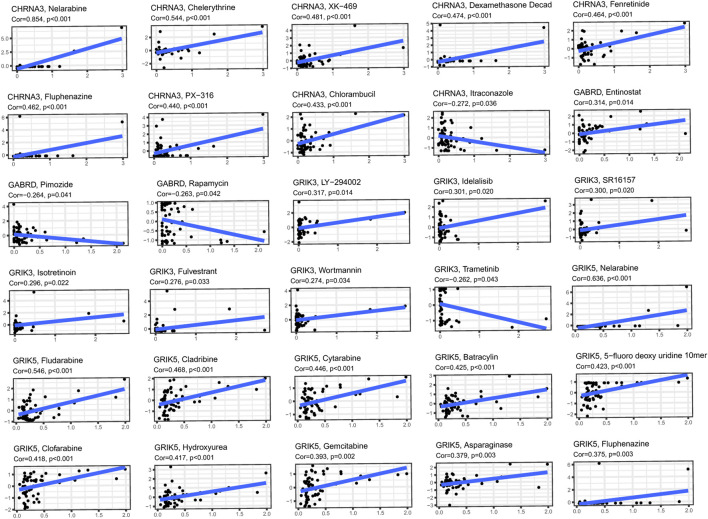
Investigating the correlation between 4 hub genes and common anti-cancer drugs using the Cellminer database.

We obtained gene expression and drug sensitivity data from CellMiner and excluded drugs without clinical trials or FDA approval and calculated the correlation coefficient between hub genes expression and drug sensitivity (Figure 11). The CHRNA3, GABRD, GRIK3 and GRIK5 gene are associated with the sensitivity of certain anti-tumor drugs such as fluphenazine, pimozide, isotretinoin, and fludarabine. CHRNA3 was identified to increase the sensitivity of cancer cells to chemotherapeutic agents, such as chelerythrine, XK-469, and dexamethasone decad, while GABRD weakened the sensitivity of pimozide and rapamycin. These findings suggest that the expression levels of hub genes may serve as a predictor of drug sensitivity for a specific class of drugs.

### Validation of mRNA expressions of prognostic NRGs

**FIGURE 12:**
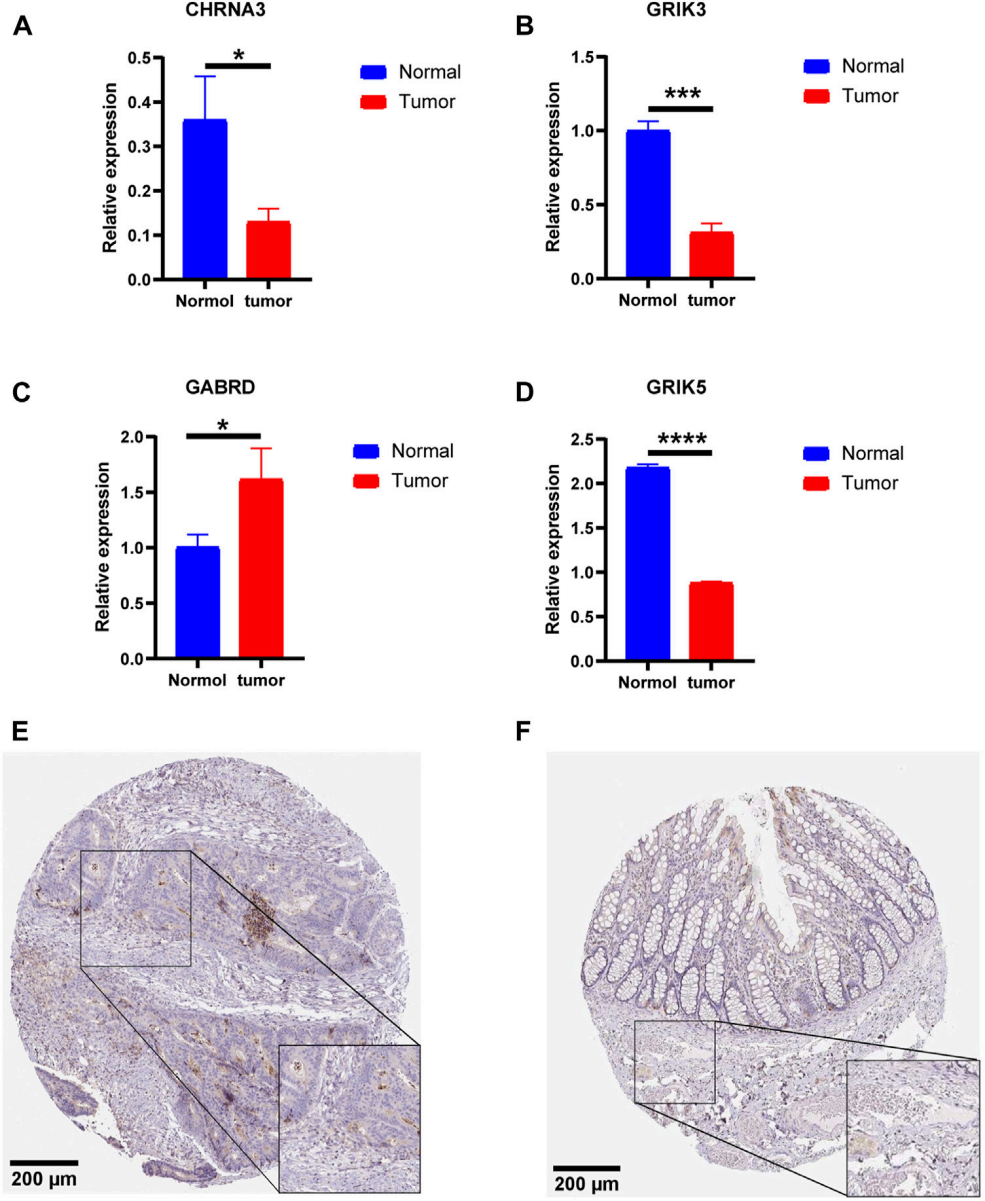
Validation of mRNA expressions of prognostic Neurotransmitter Receptor-Related Gene (NRGs). **(A–D)** The mRNA expressions of GABRD, CHRNA3, GRIK3, and GRIK5 in the normal colorectal and CRC tissues. **(E,F)** Immunohistochemistry of the GABRD in the normal and tumor groups from the HPA database. Data are shown as means ± SEM of three independent experiments. *p < 0.05, **p < 0.01, ***p < 0.001.

To confirm the significance of neurotransmitter receptor-related genes in CRC, we analyzed the differential mRNA levels of the four independent prognostic genes in the normal colorectal tissues and CRC tissues. Results revealed that, compared to normal tissues, the expression of CHRNA3, GRIK3, and GRIK5 is decreased in tumor tissues, while GABRD is highly expressed in tumor tissues, consistent with our bioinformatics analysis results (Figures 12A–C). Furthermore, we investigated the protein expression of GABRD through the HPA database. The outcomes revealed elevated protein levels of GABRD in CRC tissues (Figure 12D).

## Discussion

Neurotransmitters are traditionally known as nerve-secreted substances that modulate excitatory or inhibitory neuronal functions by binding to specific receptors. And our understanding of the regulatory role of the neurotransmitter system in tumor initiation and progression continues to advance. Neurotransmitters exhibit varying effects on the numerous functions of cancer cells, endothelial cells, and immune cells across various human cancer types. The aberrant expression of neurotransmitter signaling genes in colorectal cancer underscores the potential of neurotransmitters to enhance tumor growth and metastasis by stimulating processes such as cell proliferation, migration, invasion, and angiogenesis. In addition, neurotransmitters can influence immune cells and endothelial cells in the tumor microenvironment, fostering inflammation and contributing to the advancement of tumor growth (Battaglin et al., 2022). Nevertheless, the precise impact of various neurotransmitter receptors on colorectal cancer progression remains poorly understood. Therefore, we have identified a signature consisting of four genes associated with neurotransmitter receptors: CHRNA3, GABRD, GRIK3, and GRIK5 to predict prognosis and treatment response in CRC patients.

In our study, we employed WGCNA analysis and identified 17 modules to help explore the characteristic relationship between neurotransmitter scores and gene clusters. Additionally, we utilized machine learning algorithms to enhance the accuracy of biomarker screening. Three machine learning algorithms (LASSO logistic, SVM-RFE and RF) were primarily utilized to screen feature variables and establish the best classification model. As a result, we identified CHRNA3, GABRD, GRIK3, and GRIK5 as biomarkers by combining the machine learning algorithm and WGCNA. And these biomarkers were well-validated in the external validation cohorts.

CHRNA3 is a member of the nicotinic acetylcholine receptor (nAChRs) gene cluster, which serves as the “gateways” through which nicotine exerts its effects on the brain (Ware et al., 2012). PPI analysis reveals that CHRNA3 has a solid physical interaction with CHRNA5 and CHRNB4. Previous studies have reported that variations in CHRNA3-A5-B4 are independently and additively associated with increased cigarette consumption, nicotine dependence, and lung cancer risk (Chmielowiec et al., 2022). Despite the absence of evidence on the role of CHRNA3 in CRC progression, we have identified, for the first time, that high CHRNA3 expression is associated with poor prognosis in CRC patients. Gamma-aminobutyric acid (GABA) is the principal inhibitory neurotransmitter in the adult mammalian central nervous system. Its receptors, expressed in various tumor tissues, play a crucial role in regulating tumor cell proliferation and migration (Joseph et al., 2002; Kanbara et al., 2018; Jiang et al., 2020a; Zhang et al., 2023). The γ-aminobutyric acid type A receptor *d* subunit (GABRD), encoded in the human chromosome 1p36 region, has yet to be fully elucidated regarding its involvement in cancers (Zhang et al., 2019). Recently, Wu et al. conducted a study that found enhanced expression of GABRD to be predictive of poor prognosis in CRC patients (Yan et al., 2020), consistent with our results. And Huang et al. confirmed that GABRD receptors indicated by T cells directly inhibit CD8^+^ T cells by participating in signal regulation (Huang et al., 2022). Glutamate ionotropic receptor kainate type subunit 3 (GRIK3) and glutamate receptor ionotropic kainate-5 (GRIK5) are members of the glutamate kainate receptor family and play crucial roles in the neuroactive ligand-receptor interaction pathway (Fang et al., 2021; Minoza et al., 2022). There is compelling evidence suggesting that GRIK3 participates in cancer progression. For example, GRIK3 was reported to mediate the function of CircRNA and promote the proliferation and metastasis of colon cancer cells (Fang et al., 2021). Xiao et al. found that GRIK3 promotes epithelial-mesenchymal transition in breast cancer cells by regulating SPDEF/CDH1 signaling (Xiao et al., 2019). Furthermore, GRIK5 has been identified as a potential biomarker for melanoma metastatic progression (Minoza et al., 2022). Although limited research exists on the relationship between GRIK5 expression and colorectal cancer (CRC), our study fills this gap by identifying a significant association between high GRIK5 expression and CRC progression.

Functional enrichments were performed to gain insights into the biological processes in which these hub genes may be involved. Our results suggest that these hub genes may play critical roles in cellular metabolic processes, particularly in organic acid, inorganic acid, and lipid metabolism. It is well-established that the hostile tumor microenvironment surrounding cancer cells drives metabolic changes that impact tumorigenesis and metastatic potential. Previous studies have validated the prominent status of lipid metabolism in cancer progression. Moreover, targeting dysfunctional lipid metabolism has shown promising results as an approach to impede tumor growth (Bian et al., 2021). Amino acids also represent a crucial aspect of the tumor microenvironment, which can significantly affect cancer cell metabolism and overall tumor development (Stepka et al., 2021). Our findings suggest that these biomarkers may contribute to the construction of a TME that favors tumor development. Thus, the current study investigated the correlation between tumor infiltrating cells and neurotransmitter receptor-related gene prognostic signatures. Using the CIBERSORT algorithm, we comprehensively evaluated the abundance and infiltration of twenty-two immune cells in COAD patients. The results showed that the high expression of gene signature groups exhibits elevated levels of immunosuppressive cells, such as Tregs and macrophages M0, and low infiltration of anti-tumor cells, including CD4^+^ and dendritic cells. Previous studies have demonstrated that the significant infiltration of M0 macrophages in the tumor microenvironment may predict poor prognosis (Zheng et al., 2021), while Tregs can induce immune tolerance and facilitate immune escape and tumor metastasis (Bauer et al., 2014). These findings are consistent with our study, supporting that high expression of hub genes with increased immunosuppressive cell infiltration is associated with poor prognosis in COAD patients. The B cell populations in the TME exhibit significant heterogeneity in surface immunophenotype and function (Downs-Canner et al., 2022). Memory B cells are found in higher numbers in tumors than peripheral blood, accounting for 34% of B cells in tumors compared to 14% in peripheral blood, regardless of tumor grade. Interestingly, patients who respond to immune checkpoint inhibition therapy exhibit increased memory B cells, CXCR3+ cells, and germinal center-like B cells in the TME (Helmink et al., 2020). Here, we found a positive association between hub genes and naive B cells, indicating that the neurotransmitter receptor-related gene signature may serve as a predictive marker for the effectiveness of immunotherapy in CRC patients.

Stromal cells and endothelial cells are another important component of TME. Interestingly, in the current study, increased expression of CHRNA3, GRIK3, and GRIK5 was highly associated with stromal cells, especially CAFs. CAFs play a critical role in CRC progression and are instrumental in shaping the tumor-promoting immune microenvironment (Kobayashi et al., 2022). Additionally, endothelial cells have been identified as one of the primary sources of CAFs and play a vital role in promoting tumor metastasis (Yan et al., 2020). Notably, our results demonstrated that increased GABRD expression was highly associated with endothelial cells.

Based on the neurotransmitter receptor-related gene prognostic signatures, a prognostic nomogram was developed. And We have validated the accuracy of the nomogram through calibration plots and decision curve analysis, which supports its potential as a valuable instrument for personalized risk management. We also utilized the CellMiner database to examine the relationship between FDA-approved drugs and these four targets. Our analysis of drug sensitivity revealed that the expression of CHRNA3, GABRD, GRIK3, and GRIK5 in cancer cells significantly impacted their response to chemotherapy. Our discoveries could provide novel insights into the appropriate selection of drugs, offering guidance for forthcoming studies in oncology. Finally, we performed a simple validation of the expression of hub genes in CRC tissues and found that, compared to normal tissues, the mRNA levels of CHRNA3, GRIK3, and GRIK5 were decreased. CHRNA3, GRIK3, and GRIK5 are primarily expressed in connective tissue. Due to the predominance of tumor cells in tumor tissues, they are downregulated in tumor tissues compared to normal tissues. Both immunohistochemistry and RT-PCR confirmed the high expression of GABRD in tumor tissues. Interestingly, among these four hub genes, three of them exhibit relatively lower expression in colorectal cancer tissue compared to normal tissue. This observation indeed presents a complex and multifaceted biological phenomenon. We suggest that gene function can be independent of expression levels. The phenomenon of oncogene downregulation in tumor tissues contradicts common expectations, which generally predict higher expression levels of cancer genes. We delved into the literature and found similar instances where gene expression is influenced by various mechanisms, such as: The B-cell lymphoma 2 (BCL-2) family of proteins regulates apoptosis in normal cells. In various cancers, increased expression of BCL-2 protein is associated with enhanced drug resistance of tumor cells, although in some cases, its expression may be lower in tumor tissues compared to the surrounding normal tissues (Adams and Cory, 2007). And Hexokinase 2 (HK2) is involved in glucose metabolism in normal tissues, while in cancer cells, increased activity of HK2 is associated with cancer survival and growth, even if its expression levels are lower than in normal tissues, representing the role of metabolic reprogramming in tumors (Patra and Hay, 2014). Despite these genes being less commonly discussed, their existence and paradoxical behavior in cancer biology are well-documented. To address the question of why these three genes are expressed at lower levels in colorectal cancer tissue but are associated with a poorer prognosis in patients with high expression, further investigations in proteomics and epigenetics are required.

Though we have identified a significant gene signature in CRC based on the NRGs, there were some limitations. Firstly, our findings were based on public databases, and thus, it is crucial to validate these results in a prospective cohort from our hospital. Additionally, we need to investigate the functions of the hub genes implicated in CRC progression using cell lines and/or mouse models.

## Conclusion

In our study, we employed several bioinformatics approaches to identify a 4-gene signature related to neurotransmitter receptors to evaluate the prognosis of CRC patients. Our results indicated a significant association between the signature and the clinical features and immune system of colorectal cancer. Thus, the gene signature in our study could function as an independent prognostic indicator for CRC patients.

## Data Availability

The original contributions presented in the study are included in the article/Supplementary Material, further inquiries can be directed to the corresponding authors.

## References

[B1] Adams J. M. Cory S. (2007). The Bcl-2 apoptotic switch in cancer development and therapy. Oncogene 26, 1324–1337. 10.1038/sj.onc.1210220 17322918 PMC2930981

[B2] Battaglin F. Jayachandran P. Strelez C. Lenz A. Algaze S. Soni S. (2022). Neurotransmitter signaling: a new frontier in colorectal cancer biology and treatment. Oncogene 41, 4769–4778. 10.1038/s41388-022-02479-4 36182970 PMC10591256

[B3] Bauer C. A. Kim E. Y. Marangoni F. Carrizosa E. Claudio N. M. Mempel T. R. (2014). Dynamic Treg interactions with intratumoral APCs promote local CTL dysfunction. J. Clin. Invest. 124, 2425–2440. 10.1172/JCI66375 24812664 PMC4089459

[B4] Bian X. Liu R. Meng Y. Xing D. Xu D. Lu Z. (2021). Lipid metabolism and cancer. J. Exp. Med. 218, e20201606. 10.1084/jem.20201606 33601415 PMC7754673

[B5] Brenner H. Chen C. (2018). The colorectal cancer epidemic: challenges and opportunities for primary, secondary and tertiary prevention. Br. J. Cancer 119, 785–792. 10.1038/s41416-018-0264-x 30287914 PMC6189126

[B6] Cervantes-Villagrana R. D. Albores-Garcia D. Cervantes-Villagrana A. R. Garcia-Acevez S. J. (2020). Tumor-induced neurogenesis and immune evasion as targets of innovative anti-cancer therapies. Signal Transduct. Target Ther. 5, 99. 10.1038/s41392-020-0205-z 32555170 PMC7303203

[B7] Chin C. H. Chen S. H. Wu H. H. Ho C. W. Ko M. T. Lin C. Y. (2014). cytoHubba: identifying hub objects and sub-networks from complex interactome. BMC Syst. Biol. 8 (4), S11. 10.1186/1752-0509-8-S4-S11 25521941 PMC4290687

[B8] Chmielowiec K. Chmielowiec J. Stronska-Pluta A. Trybek G. Smiarowska M. Suchanecka A. (2022). Association of polymorphism CHRNA5 and CHRNA3 gene in people addicted to nicotine. Int. J. Environ. Res. Public Health 19, 10478. 10.3390/ijerph191710478 36078193 PMC9517777

[B9] Ding X. Liu H. Yuan Y. Zhong Q. Zhong X. (2022). Roles of GFPT2 expression levels on the prognosis and tumor microenvironment of colon cancer. Front. Oncol. 12, 811559. 10.3389/fonc.2022.811559 35330716 PMC8940194

[B10] Downs-Canner S. M. Meier J. Vincent B. G. Serody J. S. (2022). B cell function in the tumor microenvironment. Annu. Rev. Immunol. 40, 169–193. 10.1146/annurev-immunol-101220-015603 35044794

[B11] Edwards B. K. Noone A. M. Mariotto A. B. Simard E. P. Boscoe F. P. Henley S. J. (2014). Annual Report to the Nation on the status of cancer, 1975-2010, featuring prevalence of comorbidity and impact on survival among persons with lung, colorectal, breast, or prostate cancer. Cancer 120, 1290–1314. 10.1002/cncr.28509 24343171 PMC3999205

[B12] Fang G. Wu Y. Zhang X. (2021). CircASXL1 knockdown represses the progression of colorectal cancer by downregulating GRIK3 expression by sponging miR-1205. World J. Surg. Oncol. 19, 176. 10.1186/s12957-021-02275-6 34127015 PMC8204566

[B13] Favoriti P. Carbone G. Greco M. Pirozzi F. Pirozzi R. E. Corcione F. (2016). Worldwide burden of colorectal cancer: a review. Updat. Surg. 68, 7–11. 10.1007/s13304-016-0359-y 27067591

[B14] Guo L. Wang Z. DU Y. Mao J. Zhang J. Yu Z. (2020). Random-forest algorithm based biomarkers in predicting prognosis in the patients with hepatocellular carcinoma. Cancer Cell Int. 20, 251. 10.1186/s12935-020-01274-z 32565735 PMC7302385

[B15] Helmink B. A. Reddy S. M. Gao J. Zhang S. Basar R. Thakur R. (2020). B cells and tertiary lymphoid structures promote immunotherapy response. Nature 577, 549–555. 10.1038/s41586-019-1922-8 31942075 PMC8762581

[B16] Huang D. Wang Y. Thompson J. W. Yin T. Alexander P. B. Qin D. (2022). Cancer-cell-derived GABA promotes beta-catenin-mediated tumour growth and immunosuppression. Nat. Cell Biol. 24, 230–241. 10.1038/s41556-021-00820-9 35145222 PMC8852304

[B17] Jiang S. H. Hu L. P. Wang X. Li J. Zhang Z. G. (2020a). Neurotransmitters: emerging targets in cancer. Oncogene 39, 503–515. 10.1038/s41388-019-1006-0 31527667

[B18] Jiang S. H. Zhang X. X. Hu L. P. Wang X. Li Q. Zhang X. L. (2020b). Systemic regulation of cancer development by neuro-endocrine-immune signaling network at multiple levels. Front. Cell Dev. Biol. 8, 586757. 10.3389/fcell.2020.586757 33117814 PMC7561376

[B19] Joseph J. Niggemann B. Zaenker K. S. Entschladen F. (2002). The neurotransmitter gamma-aminobutyric acid is an inhibitory regulator for the migration of SW 480 colon carcinoma cells. Cancer Res. 62, 6467–6469. 12438237

[B20] Kanbara K. Otsuki Y. Watanabe M. Yokoe S. Mori Y. Asahi M. (2018). GABA(B) receptor regulates proliferation in the high-grade chondrosarcoma cell line OUMS-27 via apoptotic pathways. BMC Cancer 18, 263. 10.1186/s12885-018-4149-4 29514603 PMC5842535

[B21] Keum N. Giovannucci E. (2019). Global burden of colorectal cancer: emerging trends, risk factors and prevention strategies. Nat. Rev. Gastroenterol. Hepatol. 16, 713–732. 10.1038/s41575-019-0189-8 31455888

[B22] Kobayashi H. Gieniec K. A. Lannagan T. R. M. Wang T. Asai N. Mizutani Y. (2022). The origin and contribution of cancer-associated fibroblasts in colorectal carcinogenesis. Gastroenterology 162, 890–906. 10.1053/j.gastro.2021.11.037 34883119 PMC8881386

[B23] Kuol N. Davidson M. Karakkat J. Filippone R. T. Veale M. Luwor R. (2022). Blocking muscarinic receptor 3 attenuates tumor growth and decreases immunosuppressive and cholinergic markers in an orthotopic mouse model of colorectal cancer. Int. J. Mol. Sci. 24, 596. 10.3390/ijms24010596 36614038 PMC9820315

[B24] Langfelder P. Horvath S. (2008). WGCNA: an R package for weighted correlation network analysis. BMC Bioinforma. 9, 559. 10.1186/1471-2105-9-559 PMC2631488 19114008

[B25] Lin D. Fan W. Zhang R. Zhao E. Li P. Zhou W. (2021). Molecular subtype identification and prognosis stratification by a metabolism-related gene expression signature in colorectal cancer. J. Transl. Med. 19, 279. 10.1186/s12967-021-02952-w 34193202 PMC8244251

[B26] Lin X. Yang F. Zhou L. Yin P. Kong H. Xing W. (2012). A support vector machine-recursive feature elimination feature selection method based on artificial contrast variables and mutual information. J. Chromatogr. B Anal. Technol. Biomed. Life Sci. 910, 149–155. 10.1016/j.jchromb.2012.05.020 22682888

[B27] Li T. Fu B. Zhang X. Zhou Y. Yang M. Cao M. (2021). Overproduction of gastrointestinal 5-HT promotes colitis-associated colorectal cancer progression via enhancing NLRP3 inflammasome activation. Cancer Immunol. Res. 9, 1008–1023. 10.1158/2326-6066.CIR-20-1043 34285037

[B28] Li Z. Wang Q. Huang X. Yang M. Zhou S. Li Z. (2023). Lactate in the tumor microenvironment: a rising star for targeted tumor therapy. Front. Nutr. 10, 1113739. 10.3389/fnut.2023.1113739 36875841 PMC9978120

[B29] Minoza J. M. A. Rico J. A. Zamora P. R. F. Bacolod M. Laubenbacher R. Dumancas G. G. (2022). Biomarker discovery for meta-classification of melanoma metastatic progression using transfer learning. Genes (Basel) 13, 2303. 10.3390/genes13122303 36553569 PMC9777873

[B30] Patra K. C. Hay N. (2014). The pentose phosphate pathway and cancer. Trends Biochem. Sci. 39, 347–354. 10.1016/j.tibs.2014.06.005 25037503 PMC4329227

[B31] Ritchie M. E. Phipson B. Wu D. Hu Y. Law C. W. Shi W. (2015). Limma powers differential expression analyses for RNA-sequencing and microarray studies. Nucleic Acids Res. 43, e47. 10.1093/nar/gkv007 25605792 PMC4402510

[B32] Siegel R. L. Miller K. D. Goding Sauer A. Fedewa S. A. Butterly L. F. Anderson J. C. (2020). Colorectal cancer statistics, 2020. CA Cancer J. Clin. 70, 145–164. 10.3322/caac.21601 32133645

[B33] Sjostedt E. Zhong W. Fagerberg L. Karlsson M. Mitsios N. Adori C. (2020). An atlas of the protein-coding genes in the human, pig, and mouse brain. Science 367, eaay5947. 10.1126/science.aay5947 32139519

[B34] Stepka P. Vsiansky V. Raudenska M. Gumulec J. Adam V. Masarik M. (2021). Metabolic and amino acid alterations of the tumor microenvironment. Curr. Med. Chem. 28, 1270–1289. 10.2174/0929867327666200207114658 32031065

[B35] Sun D. Wang J. Han Y. Dong X. Ge J. Zheng R. (2021). TISCH: a comprehensive web resource enabling interactive single-cell transcriptome visualization of tumor microenvironment. Nucleic Acids Res. 49, D1420–D1430. 10.1093/nar/gkaa1020 33179754 PMC7778907

[B36] Thaker P. H. Han L. Y. Kamat A. A. Arevalo J. M. Takahashi R. Lu C. (2006). Chronic stress promotes tumor growth and angiogenesis in a mouse model of ovarian carcinoma. Nat. Med. 12, 939–944. 10.1038/nm1447 16862152

[B37] Wang H. Zhang H. Sun Z. Chen W. Miao C. (2021). GABAB receptor inhibits tumor progression and epithelial-mesenchymal transition via the regulation of Hippo/YAP1 pathway in colorectal cancer. Int. J. Biol. Sci. 17, 1953–1962. 10.7150/ijbs.58135 34131398 PMC8193267

[B38] Wang Q. Liu Y. Li Z. Tang Y. Long W. Xin H. (2023). Establishment of a novel lysosomal signature for the diagnosis of gastric cancer with *in-vitro* and *in-situ* validation. Front. Immunol. 14, 1182277. 10.3389/fimmu.2023.1182277 37215115 PMC10196375

[B39] Ware J. J. VAN Den Bree M. Munafo M. R. (2012). From men to mice: CHRNA5/CHRNA3, smoking behavior and disease. Nicotine Tob. Res. 14, 1291–1299. 10.1093/ntr/nts106 22544838 PMC3482013

[B40] Xiao B. Kuang Z. Zhang W. Hang J. Chen L. Lei T. (2019). Glutamate Ionotropic Receptor Kainate Type Subunit 3 (GRIK3) promotes epithelial-mesenchymal transition in breast cancer cells by regulating SPDEF/CDH1 signaling. Mol. Carcinog. 58, 1314–1323. 10.1002/mc.23014 30977227 PMC6618265

[B41] Yan L. Gong Y. Z. Shao M. N. Ruan G. T. Xie H. L. Liao X. W. (2020). Distinct diagnostic and prognostic values of gamma-aminobutyric acid type A receptor family genes in patients with colon adenocarcinoma. Oncol. Lett. 20, 275–291. 10.3892/ol.2020.11573 PMC7286117 32565954

[B42] Zahalka A. H. Frenette P. S. (2020). Nerves in cancer. Nat. Rev. Cancer 20, 143–157. 10.1038/s41568-019-0237-2 31974491 PMC7709871

[B43] Zhang H. Zhang L. Tang Y. Wang C. Chen Y. Shu J. (2019). Systemic screening identifies GABRD, a subunit gene of GABAA receptor as a prognostic marker in adult IDH wild-type diffuse low-grade glioma. Biomed. Pharmacother. 118, 109215. 10.1016/j.biopha.2019.109215 31545245

[B44] Zhang P. Pei S. Wu L. Xia Z. Wang Q. Huang X. (2023). Integrating multiple machine learning methods to construct glutamine metabolism-related signatures in lung adenocarcinoma. Front. Endocrinol. (Lausanne) 14, 1196372. 10.3389/fendo.2023.1196372 37265698 PMC10229769

[B45] Zhao E. Xie H. Zhang Y. (2020). Predicting diagnostic gene biomarkers associated with immune infiltration in patients with acute myocardial infarction. Front. Cardiovasc Med. 7, 586871. 10.3389/fcvm.2020.586871 33195475 PMC7644926

[B46] Zheng Y. Tian H. Zhou Z. Xiao C. Liu H. Liu Y. (2021). A novel immune-related prognostic model for response to immunotherapy and survival in patients with lung adenocarcinoma. Front. Cell Dev. Biol. 9, 651406. 10.3389/fcell.2021.651406 33816503 PMC8017122

