# Supplementary material for: Neurotransmitter receptor-related gene signature as potential prognostic and therapeutic biomarkers in colorectal cancer
Source: Front Cell Dev Biol. 2023 Nov 30;11:1202193. doi: 10.3389/fcell.2023.1202193 (PMC10720326; doi:10.3389/fcell.2023.1202193)
Supplement: Supplementary file 1 [file Table1.DOCX]

Supplementary table 1. Lists of 114 neurotransmitter receptor-related genes

| Neurotransmitter | Neurotransmitter receptor-related genes |
| --- | --- |
| Glutamate | GRIA1, GRIA2, GRIA3, GRIA4, GRID1, GRID2, GRID2IP, GRIK1, GRIK2, GRIK3, GRIK4, GRIK5, GRIN1, GRIN2A, GRIN2B, GRIN2C, GRIN2D, GRIN3A, GRIN3B, GRINA, GRIP1, GRIP2, GRIPAP1, GRM1, GRM2, GRM3, GRM4, GRM5, GRM6, GRM7, GRM8 |
| Glycine | GLRA1, GLRA2, GLRA3, GLRA4 |
| Dopamine | DRD1, DRD2, DRD3, DRD4, DRD5 |
| Histamine | HRH1, HRH2, HRH3, HRH4 |
| Epinephrine and Norepinephrine | ADRA1A, ADRA1B, ADRA1D, ADRA2A, ADRA2B, ADRA2C, ADRB1, ADRB2, ADRB3 |
| 5-HT | HTR1A, HTR1B, HTR1D, HTR1E, HTR1F, HTR2A, HTR2B, HTR2C, HTR3A, HTR3B, HTR3C, HTR3D, HTR3E, HTR4, HTR5A, HTR6, HTR7, HTR7P1 |
| Gamma aminobutyric acid (GABA) | GABBR1, GABBR2, GABRA1, GABRA2, GABRA3, GABRA4, GABRA5, GABRA6, GABRB1, GABRB2, GABRB3, GABRD, GABRE, GABRG1, GABRG2, GABRG3, GABRP, GABRQ, GABRR1, GABRR2, GABRR3 |
| Acetylcholine | CHRM1, CHRM2, CHRM3, CHRM4, CHRM5, CHRNA1, CHRNA2, CHRNA3, CHRNA4, CHRNA5, CHRNA6, CHRNA7, CHRNA8, CHRNA9, CHRNA10, CHRNB1, CHRNB2, CHRNB3, CHRNB4, CHRND, CHRNE, CHRNG |
