# Supplementary material for: Neurotransmitter receptor-related gene signature as potential prognostic and therapeutic biomarkers in colorectal cancer
Source: Front Cell Dev Biol. 2023 Nov 30;11:1202193. doi: 10.3389/fcell.2023.1202193 (PMC10720326; doi:10.3389/fcell.2023.1202193)
Supplement: Supplementary file 2 [file Table2.DOCX]

| CHRNA3 forward | 5'-TGAGCACCGTCTATTTGAGCG-3' |
| --- | --- |
| CHRNA3 reverse | 5'-TGGACACCTCGAAATGGATGAT-3' |
| GABRD forward | 5'-GCATCCGAATCACCTCCACTG-3' |
| GABRD reverse | 5'-GATGAGTAACCGTAGCTCTCCA-3' |
| GRIK3 forward | 5'-TTCGAGGCGACCAAAAAGG-3' |
| GRIK3 reverse | 5'- GGTTCACGTAGAAGGTGTCCT-3' |
| GRIK5 forward | 5'-GATCAACGGGATCATCGAGGT-3' |
| GRIK5 reverse | 5'- GTGTCCGTGGTCTCGTACTG-3' |

Supplementary table 2. Lists of the primers used in the study.
