# Supplementary material for: Neurotransmitter receptor-related gene signature as potential prognostic and therapeutic biomarkers in colorectal cancer
Source: Front Cell Dev Biol. 2023 Nov 30;11:1202193. doi: 10.3389/fcell.2023.1202193 (PMC10720326; doi:10.3389/fcell.2023.1202193)
Supplement: Supplementary file 3 [file DataSheet1.docx]

**Supplementary Figure1**


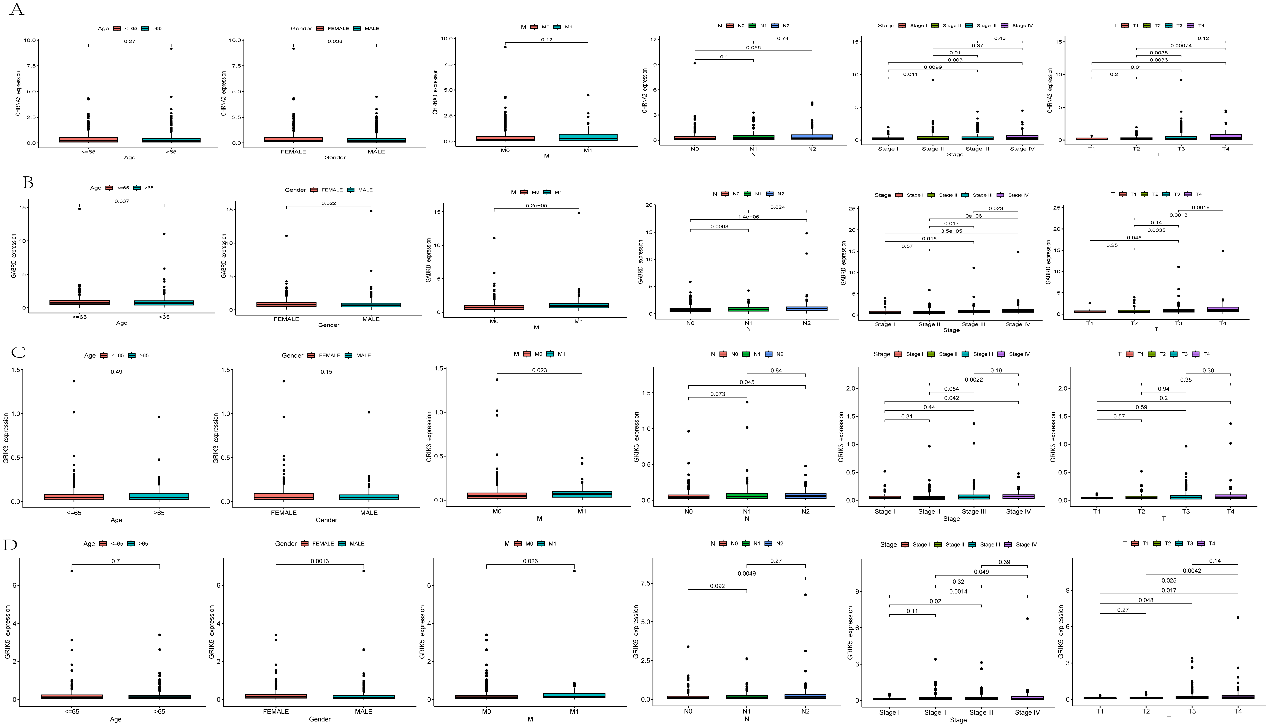


**Supplementary Figure1.** the correlation between hub genes and clinical features.

**Supplementary Figure2**


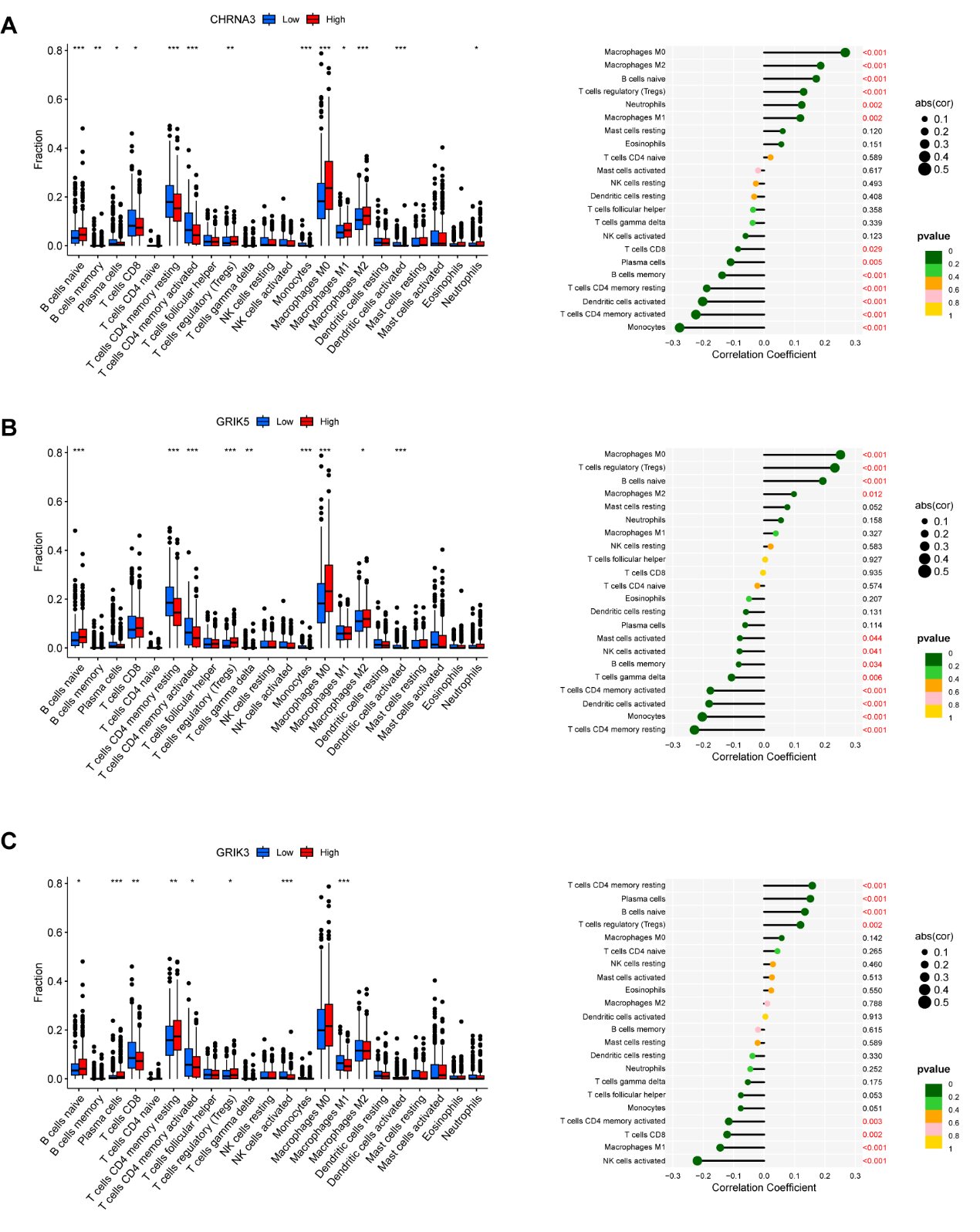


**Supplementary Figure1.** Correlation analysis between hub genes and immune cells, as well as differences in immune cells between high and low gene expression groups.
